# Human super antibody to viral RNA-dependent RNA polymerase produced by a modified Sortase self-cleave-bacteria surface display system

**DOI:** 10.1186/s12934-023-02267-z

**Published:** 2023-12-18

**Authors:** Kantaphon Glab-ampai, Kodchakorn Mahasongkram, Monrat Chulanetra, Thanatsaran Saenlom, Kanyarat Thueng-in, Nitat Sookrung, Wanpen Chaicumpa

**Affiliations:** 1 Center of Research Excellence in Therapeutic Proteins and Antibody Engineering, Department of Parasitology, Faculty of Medicine Siriraj Hospital, Mahidol University, Bangkok, 10700 Thailand; 2 School of Pathology, Translational Medicine Program, Institute of Medicine, Suranaree University of Technology, Nakhon Ratchasima, 30000 Thailand; 3 Biomedical Research Incubation Unit, Department of Research, Faculty of Medicine Siriraj Hospital, Mahidol University, Bangkok, 10700 Thailand

**Keywords:** Bacterial surface display, Cell-penetrating peptide (CPP), Human single-chain antibody variable fragment (HuscFv), RNA-dependent RNA polymerase (RdRp), RNA viruses, SARS-CoV-2, Sortase, SUMO, Super antibody

## Abstract

**Background:**

RNA-dependent RNA polymerase (RdRp) is a good target of anti-RNA virus agents; not only it is pivotal for the RNA virus replication cycle and highly conserved among RNA viruses across different families, but also lacks human homolog. Recently, human single-chain antibody (HuscFv) that bound to thumb domain of hepatitis C virus (HCV) RNA-dependent RNA polymerase (functionalized NS5B protein) was produced and engineered into cell-penetrating antibody (super antibody) in the form of cell-penetrating peptide (penetratin, PEN)-linked HuscFv (PEN-HuscFv34). The super antibody was produced and purified from inclusion body (IB) of a *pen-huscfv34*-vector-transformed *Escherichia coli*. The super antibody inhibited replication of alpha- and beta- coronaviruses, flaviviruses, and picornaviruses that were tested (broadly effective); thus, it has high potential for developing further towards a pan-anti-RNA virus agent. However, production, purification, and refolding of the super antibody molecules from the bacterial IB are laborious and hurdles to large-scale production. Therefore, in this study, Sortase-self-cleave method and bacteria surface display system were combined and modified for the super antibody production.

**Methods and results:**

BL21 (DE3) ΔA *E. coli*, a strain lacking predominant outer membrane protein (OmpA) and ion and OmpT proteases, that displayed a membrane-anchored fusion protein, i.e., chimeric lipoprotein (Lpp′)-OmpA′, SUMO, Sortase protease, Sortase cleavage site (LPET↓G) and PEN-HuscFv34-6× His was generated. The soluble PEN-HuscFv34-6× His with glycine at the N-terminus could be released from the *E. coli* surface, simply by incubating the bacterial cells in a Sortase-cleavage buffer. After centrifugation, the G-PEN-HuscFv34-6× His could be purified from the supernatant. The purified G-PEN-HuscFv34-6× retained original cell-penetrating ability (being super antibody) and the broadly effective anti-RNA virus activity of the original IB-derived-PEN-HuscFv34.

**Conclusion:**

The functionalized super antibody to RNA virus RdRp was successfully produced by using combined Sortase self-cleave and bacterial surface display systems with modification. The display system is suitable for downstream processing in a large-scale production of the super antibody. It is applicable also for production of other recombinant proteins in soluble free-folding form.

**Supplementary Information:**

The online version contains supplementary material available at 10.1186/s12934-023-02267-z.

## Background

RNA-dependent RNA polymerase (RdRp) is a versatile and functionally and structurally conserved enzyme of RNA viruses across different families. RNA-dependent RNA polymerase plays pivotal role in the RNA virus life cycle including genomic (and subgenomic) replication, transcription, and translation [1]. The polymerase usually causes a high mutation rate of the replication products [approximately 10^− 6^ to 10^− 4^ substitutions/nucleotide/cell infection (s/n/c)] due to its non-proof-reading property [2]; thus, yielding viral quasi-species in the infecting hosts [3]. Even though RdRp of different viruses may be diverse in the amino acid sequences and topological details, however, their catalytic modules are similarly surrounded by palm, fingers, and thumb domains that reminisces cupped human right-hand architecture [4]. Viral RdRp is one of the targets of broadly effective antivirals such as sofosbuvir. Recently, we produced human single-chain antibody variable fragment (HuscFv) to hepatitis C virus (HCV) RdRp (NS5B) by using phage display technology [5]. The HuscFv produced by one of phage infected-BL21 (DE3) *E. coli* clones (clone 34; HuscFv34) was molecularly linked to a cell-penetrating peptide (CPP) named penetratin (PEN), a 16-mer peptide (RQIKIWFQNRRMKWKK) from antennapedia homeodomain of the *Drosophila* transcription factor. The PEN-HuscFv34 fusion protein could traverse across mammalian cell membrane (being transbody or super antibody), bound to RdRp of the replicating HCV and inhibited the HCV NS5B polymerase activity, leading to the virus replication inhibition and the restoration of the virally suppressed-host innate immunity [5]. The super antibody also inhibited replication of the other RNA viruses that were tested, including other flaviviruses (Dengue viruses serotypes 1–4, Zika virus, Japanese encephalitis virus), picornaviruses (enterovirus-71, coxsackievirus-A16) and coronaviruses (genus *Alphacoronavirus*: Porcine Epidemic Diarrhea virus, and genus *Betacoronavirus*: SARS-CoV-2 Wuhan wild type and variants of concern) [6]. Thus, the super antibody has potential as a non-chemical pan-anti-RNA virus therapeutic agent and should be developed further towards clinical use.

The super antibody (PEN-HuscFv34) to RdRp in previous studies was produced in a laboratory scale by a transformed BL21 (DE3) *E. coli* clone 34 carrying recombinant *pen-huscfv34*-pET23b^+^ vector grown under isopropyl β-D-1-thiogalactopyranoside (IPTG) induction condition [5, 6]. The recombinant PEN-HuscFv34 was expressed as the *E. coli* inclusion body (IB). Thus, the antibody had to be purified from the *E. coli* IB using affinity resin under denaturing condition, and then refolded. The complicated IB purification and the recombinant protein refolding are hurdles to large (industrial) scale production. In this study, a simple and rapid recombinant protein production and purification method based on a bacterial surface display system [7] with calcium-dependent self-cleavage strategy [8, 9] was attuned with modification for production of soluble PEN-HuscFv34 super antibody.

In the bacterial surface display system, BL21 (DE3) ΔA *E. coli* (strain lacking gene of predominant outer membrane protein A, OmpA and two proteases, i.e., ion and OpmpT) that displayed a membrane-anchored fusion protein, from *N* to *C* termini: Lpp′-OmpA′ chimeric protein, SUMO, Sortase protease (Δ59 Sortase A), Sortase cleavage site (LPETG) and PEN-HuscFv34-6× His, was generated. The Lpp′-OmpA′ is a chimeric protein consisting of a signal sequence and the first 9 *N*-terminal amino acids of the *E. coli* membrane-anchored lipoprotein (Lpp) which is fused to a transmembrane domain (amino acid residues 46–159) of OmpA (OmpA′) [7]. This chimeric protein has been used extensively for surface display of recombinant proteins in Gram-negative bacteria [7, 9–15]. Usually, one cell of wildtype *E. coli* (with intact OmpA gene) can express as many as 100,000 OmpA molecules on the surface [7]. SUMO is a small protein (∼100 amino acid residues, ∼12 kDa in molecular mass) that covalently attaches to and detaches from other cellular proteins to modify their function. SUMO has been shown to help in increasing the expression, facilitates correct folding, as well as increasing solubility of the recombinant protein [16, 17]. Sortase is a transpeptidase, mostly found in Gram-positive bacteria. It plays an important role in attachment of the surface display proteins of Gram-positive bacteria, such as *Staphylococccus aureus* protein A, to the cell wall. It functions by cleaving specifically Leu-Thr-X-Thr-Gly (LPXTG) motif between the T and the G and catalyzes the formation of an amide bond between the carboxyl-group of T and the amino-group of the cell-wall peptidoglycan [18, 19].

To generate the BL21 (DE3) ΔA competent *E. coli* displaying the required fusion protein in this study, a DNA construct, i.e., *Lpp*′*-OmpA*′*-SUMO-*Δ*59 Sortase A-Sortase cleavage site (LPETG)-pen-huscfv34*, was inserted into a protein expression plasmid (pET28b^+^) and the recombinant vector was transformed into the BL21 (DE3) ΔA *E. coli*. After growing the transformed *E. coli*, the Lpp′-OmpA′-SUMO-Δ59 Sortase A-LPETG-PEN-HuscFv34-6× His molecules are displayed on the bacterial surface (6× His is derived from the DNA in the pET28b^+^ vector). Incubation of the transformed *E. coli* cells in a Sortase cleavage buffer containing calcium allows the Sortase to self-cleave the LPET↓G motif and release the naturally folded PEN-HuscFv34-6× His with additional glycine at the *N*-terminus (G-PEN-HuscFv34-6× His) [7, 8] into the buffer while the *N*-terminal portion of the fusion protein (Lpp′-OmpA′-SUMO-Δ59 Sortase A-LPET-) retained on the bacterial cell surface [8]. After pelleting the bacterial cells by centrifugation, the G-PEN-HuscFv34-6× His could be purified from the supernatant.

## Materials and methods

### Bacteria, plasmids, cells, viruses, and virus culture

DH5α competent *E. coli* was from Thermo Fisher Scientific, Waltham, MA, USA. BL21 (DE3) ΔA competent *E. coli* was from Addgene, Watertown, MA, USA.

Plasmids: *Lpp*′*-OmpA*′*-LPQPG-aa3h-huscfv34-pET28b*^*+*^, *Lpp*′*-OmpA*′*-SUMO-huscfv34-pET28b*^+^ and *Lpp*′*-OmpA*′*-pen-huscfv34-his-pET24a*^+^ were commercially synthesized (U_2_Bio, Seoul, South Korea). *Δ59 Sortase A-*pET28a^+^ was synthesized by GenScript (Piscataway, NJ, USA). These plasmids were available for other research in our laboratory.

African green monkey kidney (Vero and Vero E6) cells were from the American Type Culture Collection (ATCC, Manassas, VA, USA). Cells were cultured at 37 °C in humidified 5% CO_2_ incubator in Dulbecco’s modified Eagle’s medium (DMEM) (Thermo Fisher Scientific) supplemented with 10% fetal bovine serum (FBS) (Sigma*-*Aldrich, St. Louis, MO, USA), 2 mM L-alanyl-L-glutaminase dipeptide, 100 IUmL^− 1^ penicillin, and 100 μgmL^− 1^ streptomycin (complete DMEM) (Thermo Fisher Scientific).

Dengue viruses (DENV serotypes 1–4), Zika virus (ZIKV, ATCC), Porcine Epidemic Diarrhea virus (PEDV) strain P70, G2 field isolate [20], and SARS-CoV-2 (Wuhan and B1.1.529 Omicron variant) which were isolated from infected Thai COVID-19 patients were used in this study. Experiments involving live SARS-CoV-2 were performed in BSL3 facility under the approval of the Mahidol University Biosafety committee (MU2022-004). The SARS-CoV-2 viruses were propagated in Vero E6 cells while other viruses were propagated in Vero cells and titrated as described previously [6].

### Preparation of ***E. coli*** transformant displaying Lpp′-OmpA′-SUMO-Δ59 Sortase A-LPETG-PEN-HuscFv34-6× His

For preparing the *E. coli* transformant displaying the fusion protein: Lpp′-OmpA′-SUMO-Δ59 Sortase A-Sortase cleavage site (LPETG)-PEN-HuscFv34, a pET28b^+^ plasmid vector with DNA inserts (5′→ 3′) coding for amino acid residues 1–29 of *E. coli* prolipoprotein (Lpp′, NP_416192), residues 45–159 of *E. coli* OmpA (OmpA′), SUMO protein, Δ59 Sortase A, Sortase cleavage site (LPETG) and PEN-HuscFv34 was prepared. Firstly, genes coding for chimeric Lpp′-OmpA′ protein, SUMO, LPETG-PEN-HuscFv34 and Δ59 Sortase A were separately amplified from the plasmids, i.e., *Lpp*′*-OmpA*′-*LPQPG-aa3h-huscfv34-pET28b*^+^, *Lpp*′*-OmpA*′*-SUMO-huscfv34-pET28b*^+^, *Lpp*′*-OmpA*′*-pen-huscfv34-his-*pET24a^+^ and *Δ59 Sortase A-*pET28a^+^, respectively. The DNA amplicons and the pET28b^+^ were assembled and ligated appropriately by using NEBuilder^®^ HiFi DNA assembly (New England Biolabs, Ipswich, MA, USA). The *Lpp*′*-OmpA*′*-SUMO-Δ59 Sortase A-LPETG-pen-huscfv34*-pET28b^+^ recombinant vector was transformed to DH5α competent cells by heat-shock transformation method. The transformed DH5α bacteria were spread on Lennox (LN) agar plate containing 50 μgmL^− 1^ kanamycin (LN-K agar) and the plate was incubated at 37 °C overnight. The transformed *E. coli* that grew on the agar were screened for those that carried the *Lpp*′*-OmpA*′*-SUMO-Δ59 Sortase A-LPETG-pen-huscfv34*-pET28b^+^ by direct colony PCR using T7 promoter and terminator primers. The PCR thermal cycles were initial denaturation at 95 °C for 10 min followed by 30 cycles of denaturation at 95 °C for 30 s, annealing at 55 °C for 30 s and extension at 72 °C for 2 min, and final extension at 72 °C for 10 min. An *E. coli* colony positive for amplicon at ∼ 2000 bp (size of the *Lpp*′*-OmpA*′*-SUMO-Δ59 Sortase A-LPETG-pen-huscfv34-6×* *his*) was grown in LN broth containing 50 μgmL^− 1^ kanamycin (LN-K broth) to log phase. Plasmids were extracted from the bacterial cells using FavorPrep™ plasmid extraction mini kit (Favorgen Biotech Corp., Taiwan) and verified by Sanger sequencing (ATGC, Ward Medics, Bangkok, Thailand). The verified plasmid was transformed to OmpA and ion and OmpT proteases deficient BL21 (DE3) ΔA competent *E. coli*. The transformed BL21 (DE3) ΔA *E. coli* was grown in LN-K broth at 37 °C with shaking aeration (250 rpm) overnight. The overnight culture (500 μL) was added to 50 mL of fresh LN-K broth and grown further until the OD 600 nm was 0.3–0.5. Then, isopropyl-β-D-thiogalactoside (IPTG) was added to the culture to a final concentration of 1 mM, and the bacteria were grown at 37 °C with shaking (250 rpm) for 5 h. The culture was centrifuged (4000 × *g*, 4 °C, 30 min). The bacterial pellet was washed three times with PBS, fixed with 4% paraformaldehyde art room temperature (RT, 25 ± 2 °C) for 20 min, washed with PBS, stained with mouse mAb to HuscFv34 and goat anti-mouse Ig-Alexa Fluor 488, at RT for 1 h. The bacteria were washed with PBS and stained with DAPI to indicate nuclei. The stained cells were subjected to flow cytometric analysis (BD FACSymphony A1, BD LSRFortessa™, BD, Franklin Lakes, New Jersey, USA) for checking surface displayed Lpp′-OmpA′-SUMO-Δ59 Sortase A-LPETG-PEN-HuscFv34-6× His.

### Preparation of monoclonal antibodies to HuscFv34

Monoclonal antibodies (mAbs) to HuscFv34 were prepared for use as a tracer of the HuscFv34 as described previously [20]. Mouse experiments were approved by Siriraj Animal Care and Use committee of the Faculty of Medicine Siriraj Hospital, Mahidol University, Bangkok (Si-ACUC No. 012/2564).

HuscFv34 was prepared from *E. coli* clone 34 and purified as described previously [5]. Three female BALB/c mice, 7–9 weeks old (Nomura, Bangkok, Thailand) were bled to collect pre-immune serum samples before injecting individually and intraperitoneally with 10 μg of purified HuscFv34 mixed with Imject Alum adjuvant (Thermo Fisher Scientific) at a ratio of 3:1 (v/v) in a total volume of 200 μL. Two booster doses of the same immunogen, same amount, and same route were given to each primed mice at two weeks-intervals. Two weeks after the third immunization, the immunized mice bled, and serum samples were checked for antibody titers to the HuscFv34 protein by indirect ELISA. An immune mouse with the highest antibody titer to HuscFv34 was given an intravenous dose of 20 μg of purified HuscFv34 in 100 μL Dulbecco’s phosphate-buffered saline (DPBS, Thermo Fisher Scientific). Three days later, the mouse was euthanized, and spleen was collected. Single spleen cells were prepared, washed, and resuspended with ClonaCell™-HYClonaCell™-HY Medium B serum-free medium (Stemcell technologies, Vancouver, Canada). The cells were mixed with log phase grown P3×-63-Ag8.653 myeloma cells at a ratio of ~ 5:1. The cell mixture was then centrifuged at 400 × *g* at 25 °C for 10 min. After removal of supernatant, 1 mL of ClonaCell™-HY PEG (Stemcell technologies) was slowly added to the cell pellet. Fused cells were then added with ClonaCell™-HY Medium C (Stemcell technologies) and kept at 37 °C in 5% CO_2_ humidified incubator for 16–20 h. The cells were collected by centrifugation 400 × *g* at 25 °C for 10 min; the cell pellet was reconstituted in ClonaCell™-HY Medium C and transferred to semi-solid ClonaCell™-HY Medium D containing hypoxanthine, aminopterin and thymidine (HAT) (Stemcell technologies). Then the cell mixture was slowly drawn and plated into 10 × 100 mm Petri dishes. The culture dishes were kept at 37 °C in a 5% CO_2_ humidified incubator in a sterile moist chamber for 10–14 days. Single colonies were randomly picked and transferred separately into wells of 96-well plate containing 200 μL of ClonaCell-HY medium E (Stemcell technologies) and cultured at 37 °C in a 5% CO_2_ humidified incubator for 3–4 days. The hybridoma culture supernatants were collected separately and screened for mAbs to HuscFv34 by indirect ELISA using HuscFv34 as antigen and homogenate of original BL21 (DE3) competent *E. coli* as control antigen to coat separate ELISA wells (1 μg per well). Then the HuscFv34-bound mAb of a selected hybridoma clone was subjected to isotyping (Thermo Fisher Scientific).

For production and purification of mAb to HuscFv34 in a large scale, the selected mouse hybridoma clone was expanded in ClonaCell-HY medium E at 37 °C in a 5% CO_2_ humidified incubator. Subsequently, the cells were adapted to complete RPMI-1640 medium and then to ISF-1 serum-free medium (PanBiotech, Aidenbach, Germany), respectively. Adaptation from complete RPMI-1640 medium to ISF1 serum-free medium was performed by sub-culturing the hybrid cells in increasing concentrations of ISF-1 serum-free medium and decreasing concentrations of complete RPMI-1640 medium. The FBS in the medium was gradually reduced from 20 to 0%. The adapted hybridoma cells were cultured in ISF1 serum-free medium at 37 °C in a 5% CO_2_ humidified incubator for 5–7 days. The culture supernatant was then harvested and centrifuged. The clear culture supernatant containing the mAb to HuscFv34 was subjected to Hitrap Protein G column (Cytiva, Marlborough, Massachusetts, USA) affinity chromatography for the mAb purification.

### Western blot analysis

The antigenic preparation was electrophoresed in sodium dodecyl sulfate-polyacrylamide gel (SDS-PAGE) using 4% stacking gel and 12% separating gel cast in Mini-PROTEAN Cell (Bio-Rad, Hercules, California, USA). The SDS-PAGE-separated components in the separating gels were electroblotted onto nitrocellulose membrane (NC) and the unoccupied sites on the blotted NC were blocked with 5% skim milk in Tris-buffered saline containing 0.01% Tween-20 (TBS-T) for 1 h. Excess blocking reagent was discarded; the NC was washed three times with the TBS-T and placed in a solution of mouse mAb to HuscFv34, mouse anti-His (Bio-Rad), or mouse anti-SUMO (Genscript) at RT for 1 h. The NC was washed again with the TBS-T and incubated with goat-anti-mouse Ig-alkaline phosphatase (AP) conjugate (SouthernBiotech, Birmingham, AL, USA) for 1 h. After washing with TBS-T, BCIP/NBT substrate (KPL, SeraCare, Millford, MA, USA) was used to reveal the antigen-antibody reactive bands. The NC was washed with distilled water and air-dried.

### Flow cytometry for detecting PEN-HuscFv34-6× His on the surface of transformed BL21 (DE3) ΔA competent ***E. coli***

Transformed BL21 (DE3) ΔA *E. coli* (4 × 10^8^ cells) was washed once with PBS. The bacterial pellet was fixed with 4% paraformaldehyde at RT for 30 min and washed twice with PBS before mixing with 100 μL of 1:200 of mouse mAb to HuscFv34 (clone 5F7) and kept at RT for 1 h. After centrifugation, the pellet was washed twice with 500 μL of FACS buffer (2% FBS and 0.02% sodium azide in PBS) and stained with 100 μL of 1:200 goat anti-mouse antibody-Alexa Fluor 488 (Thermo Fisher Scientific) on ice, in darkness for 1 h. After washing with FACS buffer, the pellet was stained with 200 μL of 1:250 of 4′,6-diamidino-2-phenylindole (DAPI) at RT in darkness for 30 min. The stained bacterial cells were washed and resuspended in 300 μL of 1% paraformaldehyde. The PEN-HuscFv34-6× His displayed on bacterial cell surface was determined by flow cytometry (BD LSRFortessa™, BD).

### Preparation and purification of the PEN-HuscFv34-6× His

The *Lpp*′*-OmpA*′*-SUMO-D59 Sortase A-LPETG-pen-huscfv34*-pET28b^+^-transformed-BL21 (DE3) ΔA competent *E. coli* was cultured in the LN-K broth under the optimal IPTG-induced condition and temperature. Expression of the Lpp′-OmpA′-SUMO-59 Sotase A-LPETG-PEN-HuscFv34-6× His on the bacterial cell surface was checked by flow cytometric analysis. Bacterial pellet from 1000 mL-culture was washed twice with Tris-buffer [50 mM Tris (pH 7.5) and 150 mM NaCl] and the pellet of the last wash was resuspended in a Sortase cleavage buffer [50 mM Tris (pH7.5), 150 mM NaCl, 5 mM CaCl_2_] at 1-gram bacterial wet weight to 10 mL buffer and incubated in a 37 °C water-bath. Aliquots of the preparation were taken at 0, 1, 2, 3, 4 and 5 h incubation and centrifuged (12,000 × *g*, 4 °C, 10 min); the supernatants containing the PEN-HuscFv34-6× His with additional glycine at the N-terminus (G-PEN-HuscFv34-6× His) in the clear supernatants were subjected to Western blot analysis using the mouse anti-His as a tracer.

For preparing purified G-PEN-HuscFV34-6× His, each 1 gram of *E. coli* pellet was resuspended with 10 mL Sortase cleavage buffer and incubated 37 °C water-bath for 2 h. The supernatant containing G-PEN-HuscFv34-6× His was collected, centrifuged, and purified using the Histrap column (Cytiva, 100 Results Way, Marlborough, USA). The Histrap column was washed and equilibrated with binding buffer (50 mM Tris-HCL, pH 7.5 and 150 mM NaCl) with 1 mL per min flow rate. The supernatant containing the G-PEN-HuscFv34-6× His was filtered before loading to the column using the same flow rate. After washing with 25 mL binding buffer, the G-PEN-HuscFv34-6× His was eluted with elution buffer (binding buffer containing 150 mM imidazole) into 15 fractions (1-mL fractions) and all fractions were subjected to SDS-PAGE and Coomassie Brilliant Blue G 250 (CBB) staining to verify the G-PEN-HuscFv34-6× His purity. The fractions containing purified G-PEN-HuscFv34-6× His were pooled and dialyzed against phosphate-buffered saline, pH 7.4 (PBS) for further use.

### Biocompatibility of the G-PEN-HuscFv34-6× His to mammalian cells

Biocompatibility of the G-PEN-HuscFv34-6× His to Vero and Vero E6 cells (representative of mammalian cells) was determined as described previously [6]. The cell monolayer established overnight in wells of 96-well-white plate were added with various concentrations (0.0157, 0.0313, 0.0625, 0.125, 0.25, 0.5, 1.0 and 2.0 μM) of the G-PEN-HuscFv34-6× His and incubated at 37 °C in a 5% CO_2_ incubator overnight. The culture medium alone added separately to the wells of Vero and Vero E6 cells served as negative cytotoxic controls. Cytotoxicity of the G-PEN-HuscFv34-6× His was determined using Cell Counting kit-8 (CCK-8, Dojindo Laboratories, Kaminashiki-gun, Kumamoto, Japan). After keeping the plate at 37 °C in a 5% CO_2_ incubator overnight, the CCK-8 solution containing WST-8 (10 μL) was added to individual wells of the G-PEN-HuscFv34-6× His-treated cells and controls and incubated at 37 °C for 1 h. The colorless WST-8 was reduced by dehydrogenase produced by viable cells to give rise to orange color product (formazan) which the amount [detected at OD 450 nm by using Synergy H1 microplate reader (Agilent, Santa Clara, CA, USA)] was directly proportional to the living cell number. The percentage of cell viability was calculated compared to the negative cytotoxic control.

### Determination of cell-penetrating ability of the G-PEN-HuscFv34-6× His

Vero E6 cells (1.5 × 10^5^ cells in 500 μL of complete DMEM) were seeded onto a cover slip placed in well of a 24-well-culture plate and kept at 37 °C in 5% CO_2_ incubator overnight. The fluid was discarded and the G-PEN-HuscFv34-6× His (0.5 μM) was added to the well containing the Vero E6 cells. The Vero E6 cells in medium served as control (no antibody added). After overnight incubation at 37 °C in 5% CO_2_ atmosphere, the cells were washed with PBS, fixed and permeated with 500 μL of ice-cooled methanol:acetone on ice for 15 min. After washing with PBS, the cells were blocked with 3% BSA in PBS at RT for 30 min, washed with PBS, and added with mouse mAb to HuscFv34 at RT for 1 h. After washing again with PBS, the cells were counterstained with goat anti-mouse Ig-Alexa Fluor 488 conjugate (1:200) on ice in darkness for 1 h. DAPI was used to stain nuclei. After washing, the cover slip was placed on a glass slide; the cells were mounted and observed under a confocal microscopy for intracellular G-PEN-HuscFv34-6× His.

### Inhibition of RNA virus infectivity by the G-PEN-HuscFv34-6× His (super antibody)

The experiments were performed as described previously [6]. For dengue virus (DENV) serotypes 1–4 and zika virus (ZIKV), Vero cell monolayer established overnight in wells of 24-well cell culture plates (1.5 × 10^5^ cells per well) were infected with DENV serotypes 1–4 or ZIKV at MOI 0.1 individually and kept at 37 °C in 5% CO_2_ incubator for 1 h. After removing the fluids, the infected cells were added with 500 μL of complete DMEM containing 0.5 μM G-PEN-HuscFv34-6× His or medium alone (negative inhibition control) and incubated at 37 °C in 5% CO_2_ incubator for 48 h. The virus RNA in each cell sample was quantified by real-time reverse transcription-polymerase chain reaction (qRT-PCR) [6].

The Vero cells were infected with porcine epidemic diarrhea virus (PEDV) at MOI 0.0005. The DMEM supplemented with 2 μg/mL TPCK trypsin (Thermo Scientific Pierce) containing 0.5 μM G-PEN-HuscFv34-6× His or medium alone (negative inhibition control) were added to the infected cells. The treated infected cells were incubated 37 °C in 5% CO_2_ incubator for 18 h and the viral RNAs were extracted from the cells for the PEDV RNA quantification by qRT-PCR.

All experiments using SARS-CoV-2 were conducted in BLS3 facility of the Faculty of Medicine Siriraj Hospital, Mahidol university. After infected with SARS-CoV-2 (Wuhan wide type or BA.1.1.529 Omicron variant), the infected cells were treated with 0.5 μM G-PEN-HuscFv34-6× His in complete DMEM or medium alone and incubated for 18 h. RNA was extracted from the harvested cells and the SARS-CoV-2 RNAs were determined by qRT-PCR.

### Real-time reverse transcription-polymerase chain reaction (qRT-PCR)

RNAs extracted from individual cell samples (TRIzol™ reagent; Thermo Fisher Scientific) were quantified for viral RNAs by qRT-PCR using Brilliant III Ultra-Fast SYBR® Green QRT-PCR Master Mix (Agilent Technologies) and PCR primers as described previously [6]. Each PCR reaction consisted of 10 μL of 2 × SYBR Green qRT-PCR Master Mix, 0.4 μL each of forward and reverse primers, 0.2 μL of 100 mM DTT, 1 μL of RT/RNase Block, 6 μL of nuclease free water and 2 μL of RNA template. The PCR reaction condition was 50 °C, 10 min; 95 °C, 3 min; and 40 cycles of 95 °C, 5 s and 60 °C, 5 s. The amounts of viral RNAs of the super antibody-treated groups were compared to the infected cells in medium alone. The fold differences of viral RNAs in the infected cells treated with the G-PEN-HuscFv34-6× His compared to the controls were calculated.

### Statistical analysis

GraphPad Prism version 9.3 (GraphPad Software; San Diego, CA, USA) was used to compare the results of all tests. Statistically significant difference was determined by one-way ANOVA. *p*-value of 0.05 or lower was considered statistically significant. *p* > 0.05 (ns, not significant); *p* ≤ 0.05 (*), *p* ≤ 0.01 (**), *p* ≤ 0.001 (***), *p* ≤ 0.0001 (****).

## Results

### ***Escherichia coli*** displaying Lpp’-OmpA′-SUMO-Δ59 SortaseA-LPETG-PEN-HuscFv34-6× His

**Fig. 1:**
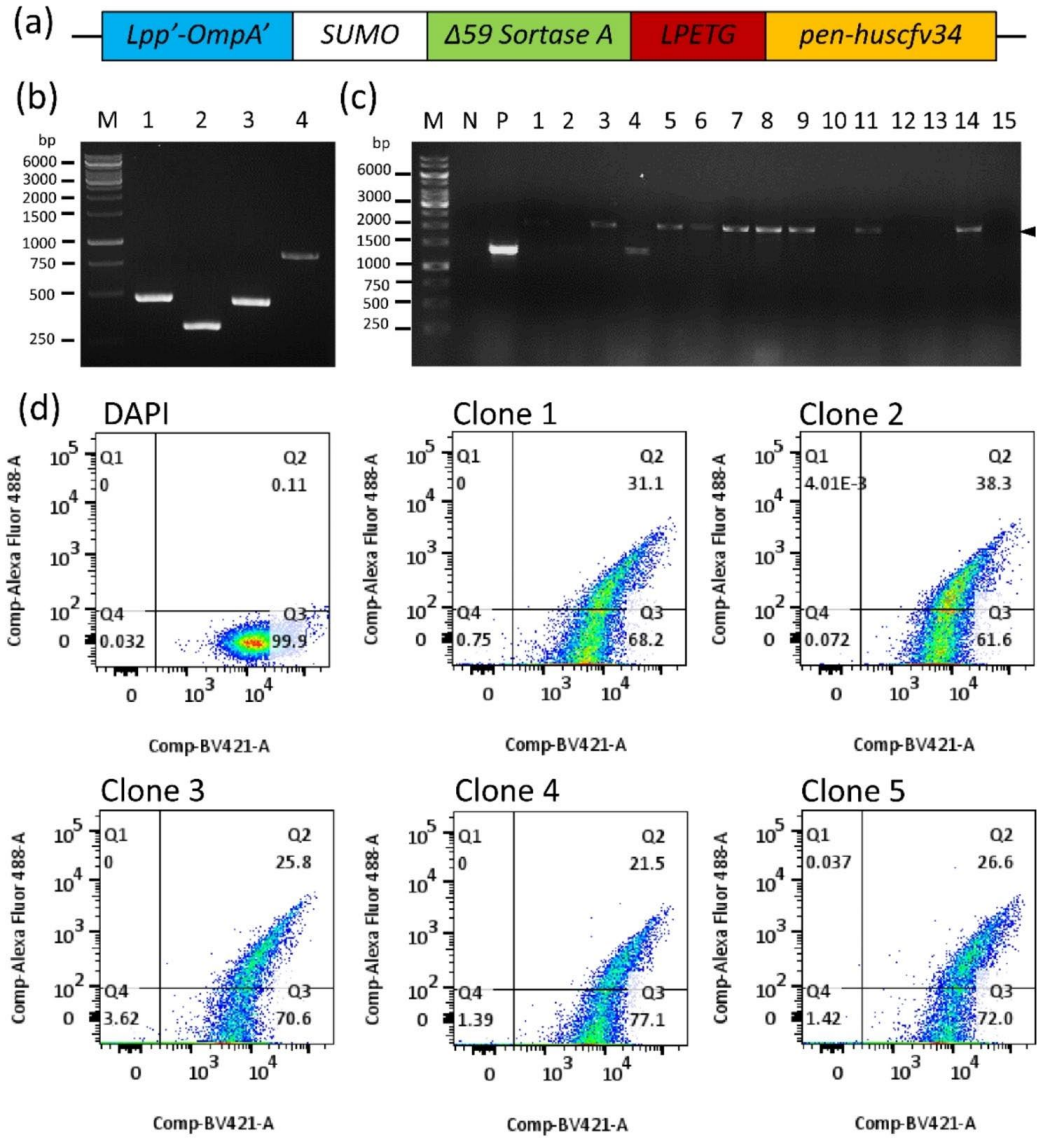
Preparation of *E. coli* transformants that displayed LPP′-OmpA′-SUMO-Δ59 Sortase A-LPETG-PEN-HuscFv34-6× His on the surface. (**a**) Diagram of a DNA construct coding for Lpp′-OmpA′-SUMO-Δ59 Sortase A-LPETG-PEN-HuscFv34. (**b**) Amplicons of *Lpp*′*-OmpA*′ (456 bp), *SUMO* (288 bp), *Δ59 Sortase A* (447 bp) and *LPETG-pen-huscfv34* (840 bp). (**c**) Amplicons of *Lpp*′*-OmpA*′-Δ*59 Sortase A-LPETG-pen-huscfv34* contig (∼2000 bp, arrowhead) in the DH5α competent *E. coli* clones 1–15 that had been transformed with *Lpp*′*-OmpA*′-Δ*59 Sortase A-LPETG-pen-huscfv34*-pET28b^+^ vector (lanes 1–15, respectively); lanes M of (b) and (c) are DNA size standard; lane N of (c) is negative DNA template control; lane P of (c) is positive DNA template control; numbers at the left of (b) and (c) are DNA sizes in bp. (**d**) Flow cytometric analysis of transformed BL21 (DE3) ΔA competent *E. coli* clones 1–5 that were stained by mouse monoclonal anti-HuscFv34 antibody and goat anti-mouse Ig-Alexa Fluor 488 conjugate for detection of Lpp′-OmpA′-SUMO-Δ59 Sortase A-LPETG-PEN-HuscFv34-6× His fusion protein on the cell surface. Cells were counter-stained by DAPI to indicate nuclei. Q1 and Q2, transformed *E. coli* cells displaying Lpp′-OmpA′-SUMO-Δ59 Sortase A-LPETG-PEN-HuscFv34-6× His fusion protein; Q3 and Q4, *E. coli* cells that did not display the fusion protein. Q2 and Q3, intact bacterial cells with nuclei; Q1 and Q4, bacterial cells without nuclei or dead cells

Diagram of a DNA construct coding for Lpp′-OmpA′-SUMO-Δ59 Sortase A-LPETG-PEN-HuscFv34 fusion protein is shown in Fig. 1a. For generating the DNA construct, genes coding for chimeric LPP′-OmpA′, SUMO, Δ59 Sortase A and LPETG-PEN-HuscFv34 were individually amplified using synthetic plasmids available in our laboratory as templates and oligonucleotide primers, as listed in Table 1. Amplicons of these genes are shown in Fig. 1b. These amplicons were assembled and ligated appropriately to pET28b^+^ plasmid by using a NEBuilder master mix. The recombinant vector was transformed into DH5α *E. coli*. Fifteen colonies of the transformed DH5α *E. coli* that grew on a selective agar plate were screened by PCR for the expected plasmid amplicons, eight colonies were positive (Fig. 1c). Plasmids extracted from four of the eight transformed DH5α *E. coli* clones were verified by Sanger sequencing; the verified plasmids were transformed into BL21 (DE3) ΔA competent *E. coli*. Five transformed BL21 (DE3) ΔA *E. coli* clones were grown in small scale and checked for the surface displayed Lpp’-OmpA′-SUMO-Δ59 Sortase A-LPETG-PEN-HuscFv34-6× His by flow cytometric analysis. Among the five clones, cells of clone 2 showed the highest percentage of surface-displayed Lpp′-OmpA′-SUMO-Δ59 Sortase A-LPETG-PEN-HuscFv34-6× His fusion protein (Fig. 1d). This clone was tested further.

**Table 1:** Oligonucleotide primers and plasmid templates used for amplification of DNA sequences coding for chimeric LPP′-OmpA′, SUMO, Δ59 Sortase A and LPETG-PEN-HuscFv34

Gene	Primer (5′ to 3′)		Plasmid template
	Forward	Reverse	
*LPP*′*-OmpA*′	GGAGATATACCATGGCGATGAAAGCTACTAAACTG	ATTGACTTCTGAGTCGGTACCAAGCTTGTCGACTC	*Lpp*′*-OmpA*′*-LPQPG-aa3h-huscfv34*-pET28b^+^
*SUMO*	GGAGTCGACAAGCTTGGTACCGACTCAGAAGTCAAT	CTGCGGTTTTGCCTGGGTACCGCCACCAATCTGTTC	*Lpp*′*-OmpA*′*-SUMO-huscfv34*-pET28b^+^
*∆59 Sortase A*	GAACAGATTGGTGGCGGTACCCAGGCAAAACCGCAG	GCCGGTCTCGGGCAGTTTCACTTCGGTTGC	*Sortase A-Del59-*pET28a^+^
*LPETG-pen-huscfv34*	GCAACCGAAGTGAAACTGCCCGAGACCGGC	TGGTGGTGCTCGAGTTATTAGTGGTGGTG	*Lpp*′*-OmpA*′*-pen-huscfv34-his*-pET24a^+^

### Mouse monoclonal antibodies (mAbs) to HuscFv34

**Fig. 2:**
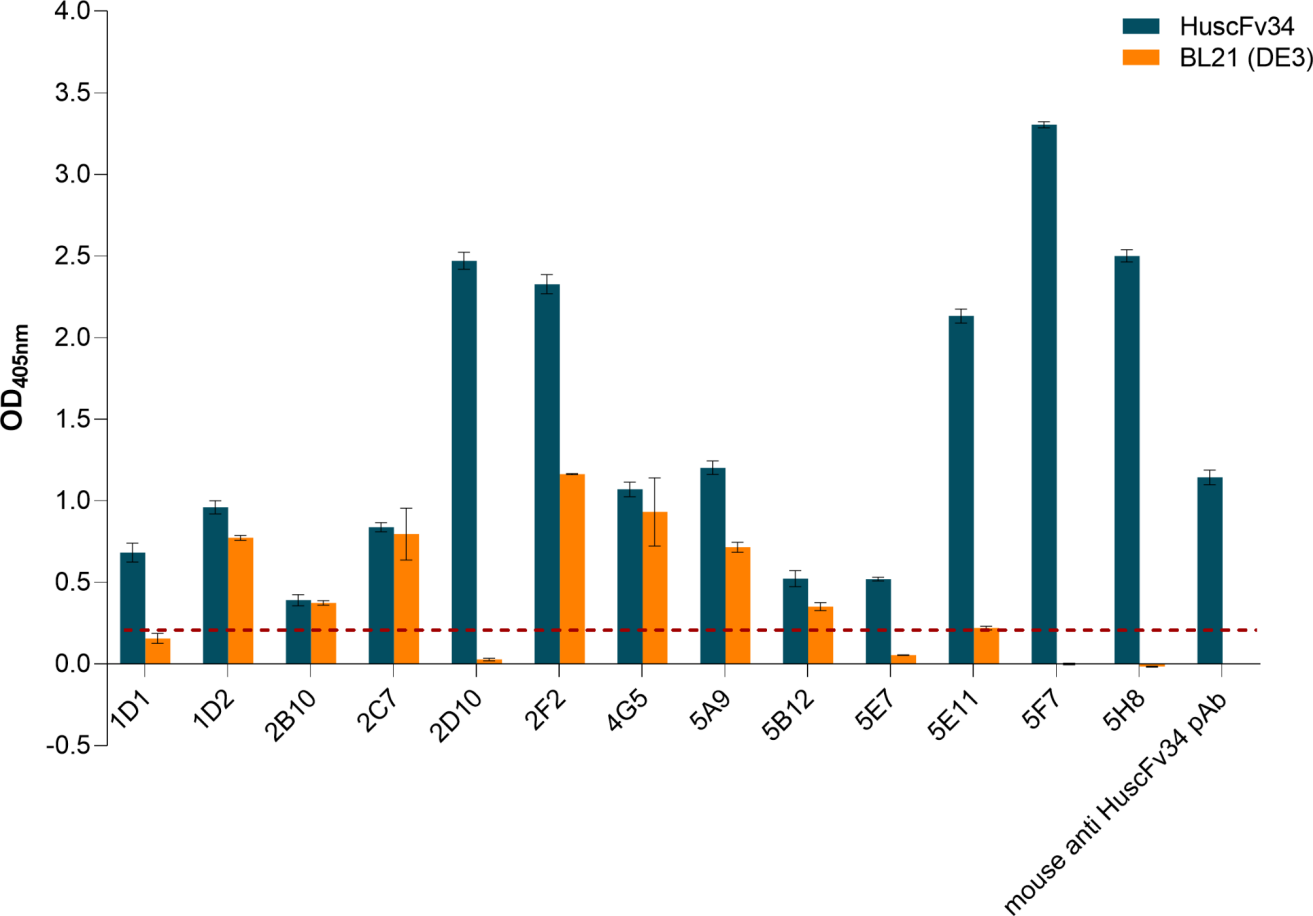
Indirect ELISA titers of mAbs in spent culture supernatants of hybridomas raised against HuscFv34. HuscFv34 (1 μg per well) was used as antigen in the indirect ELISA. Homogenate of original BL21 (DE3) *E. coli* was used as control antigen. Mouse immune serum (mouse anti-HuscFv34 pAb) at dilution 1:5000 served as positive antibody control. X axis, culture supernatant of hybridoma clones; Y axis indirect ELISA OD 405 nm against blanks (wells added with PBS instead of antibody). The dotted line is the arbitrarily cut-off level (OD 405 nm = 0.2) between positive and negative indirect ELISA results

Mouse monoclonal antibodies (mAbs) to HuscFv34 were prepared for use as a tracer of the HuscFv34 in immunoassays. To generate hybridomas secreting mAbs to the HuscFv34, splenocytes of a BALB/c mouse immunized with recombinant HuscFv34 derived from inclusion body of transformed BL21 (DE3) *E. coli* [5, 6] were fused with P3×-63-Ag8.653 mouse myeloma cells. Hybrid cells (hybridomas) were selected and nurtured to produce mAbs. Indirect ELISA titers of the mAbs secreted by different hybridoma clones at the late log phase of the hybridoma growth against the HuscFv34 and control antigen [homogenate of the original BL21 (DE3) ΔA competent *E. coli*] are shown in Fig. 2. The mAbs in the culture supernatant of clone 5F7 which produced high titer mAb (IgG-kappa isotype) specific to the HuscFv34 was purified and used subsequently as the HuscFv34 tracer.

### Preparation of G-PEN-HuscFv34-6× His from the transformed ***E. coli*** displaying Lpp′-OmpA′-SUMO-Δ59 Sortase A-LPETG-PEN-HuscFv34-6× His

**Fig. 3:**
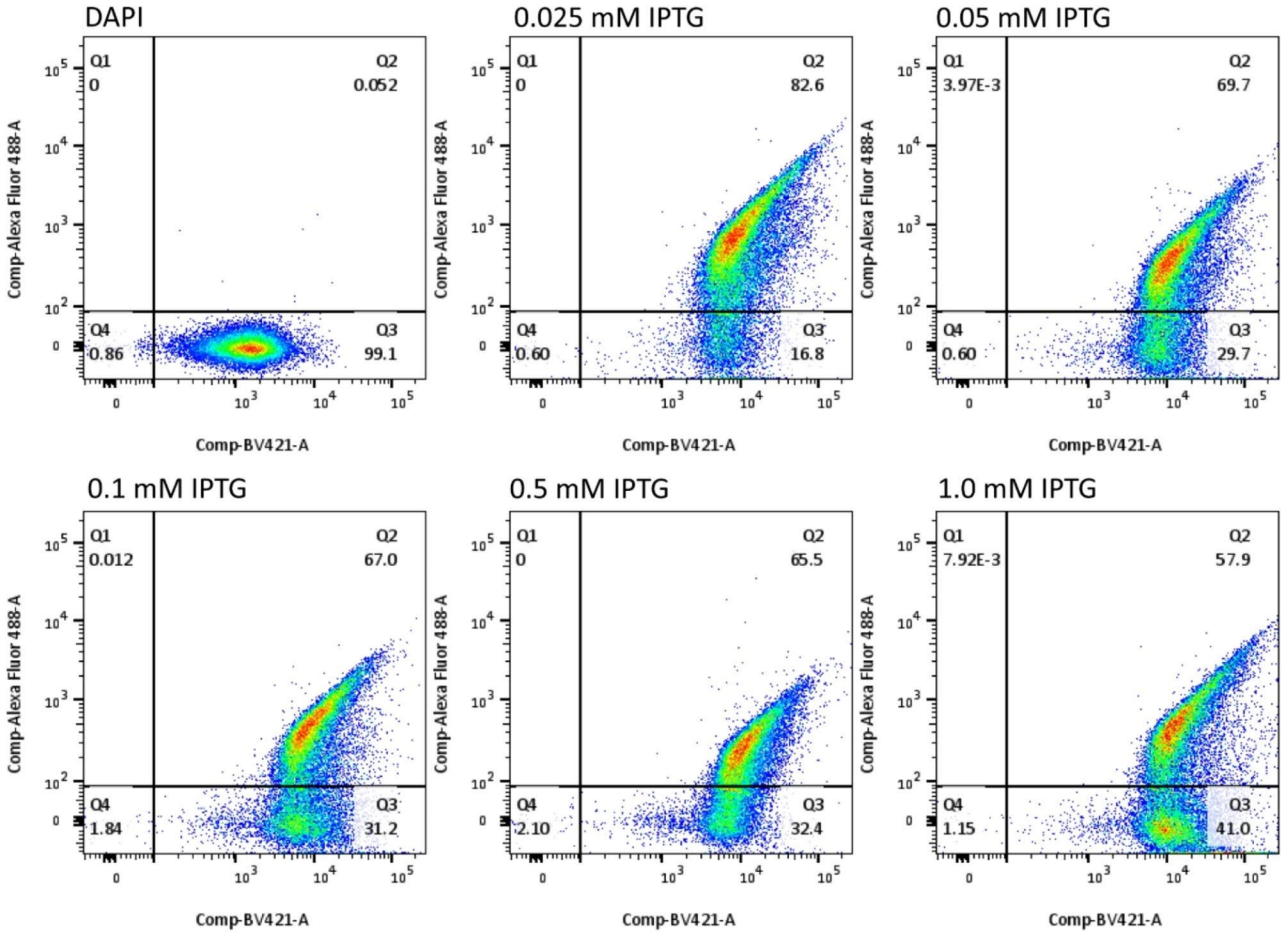
Optimal IPTG concentration in induction of LPP′-OmpA′-SUMO-Δ59 Sortase A-LPETG-PEN-HuscFv34-6× His expression on the bacterial surface. The transformed BL21 (DE3) ΔA competent *E. coli* was grown under induction of different IPTG concentrations (0.025, 0.05, 0.1, 0.5 and 1.0 mM) at 37 °C. The bacterial cells were fixed with 4% paraformaldehyde, washed, incubated with mouse mAb to HuscFv34, washed, added with goat anti-mouse Ig-Alexa Fluor 488 conjugate, washed, stained with DAPI, washed, and resuspended in 1% paraformaldehyde. The PEN-HuscFv34-6× His displayed on bacterial cells was determined by flow cytometry. Q1 and Q2, transformed *E. coli* cells displaying Lpp′-OmpA′-SUMO-Δ59 Sortase A-LPETG-PEN-HuscFv34-6× His fusion protein; Q3 and Q4, *E. coli* cells that did not display the fusion protein. Q2 and Q3, intact bacterial cells with nuclei; Q1 and Q4, bacterial cells without nuclei or dead cells

**Fig. 4:**
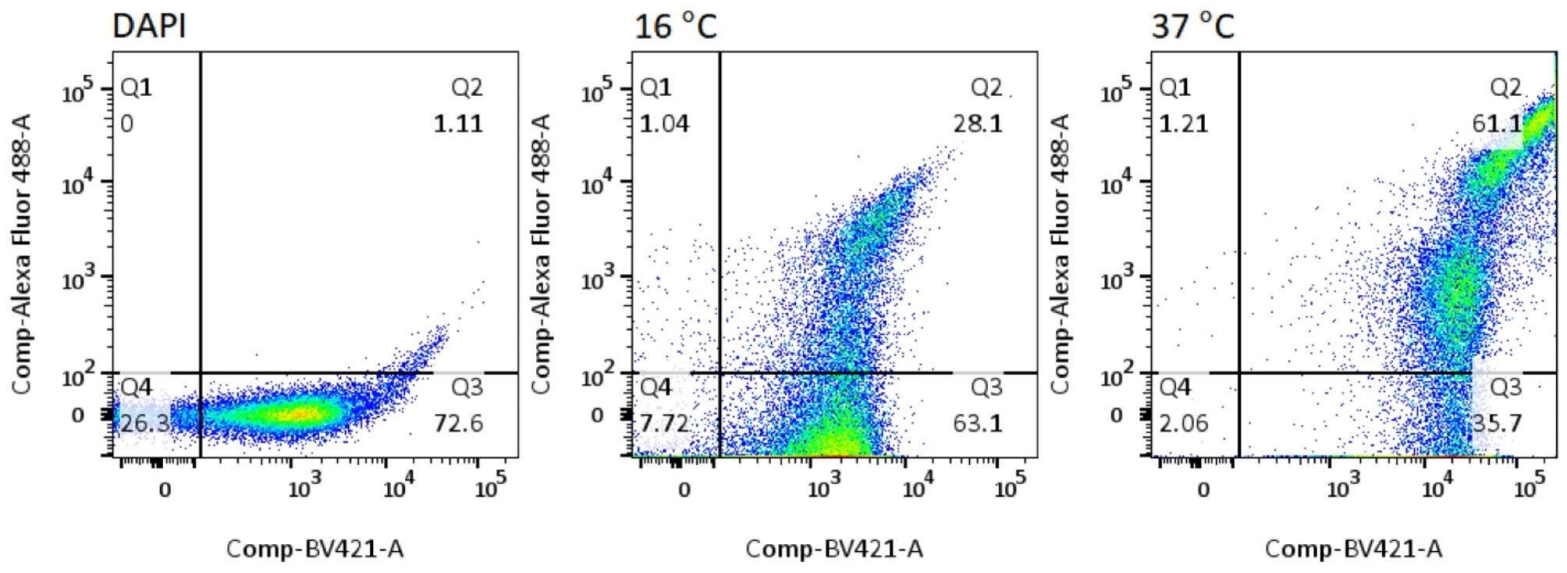
Suitable temperature for growing 0.025 mM IPTG-induced transformed bacteria to express the fusion protein. The transformed BL21 (DE3) ΔA competent *E. coli* was grown under 0.025 mM IPTG induced condition at 16 and 37 °C for 5 h. The cells were then stained as in Fig. 3 and analyzed for percent surface displayed LPP′-OmpA′-SUMO-Δ59 Sortase A-LPETG-PEN-HuscFv34-6× His by flow cytometry. The transformed bacteria cultured at 37 °C expressed higher percentage of the cells displaying the fusion protein on surface (61.1%) than when they were grown at 16 °C (28.1%)

The transformed BL 21 (DE3) ΔA competent *E. coli* was grown in small scale (10 mL LN-K broth) and induced by different concentrations of IPTG (0.025, 0.05, 0.1, 0.5 and 1.0 mM). The IPTG at 0.025 mM induced the highest percentage (82.6%) of the *E. coli* cells displaying Lpp′-OmpA′-SUMO-Δ59 Sortase A-LPETG-PEN-HuscFv34-6× His (Fig. 3). The optimal temperature for growing the *E. coli* transformant displaying the fusion protein on the surface was 37 °C as shown in Fig. 4.

**Fig. 5:**
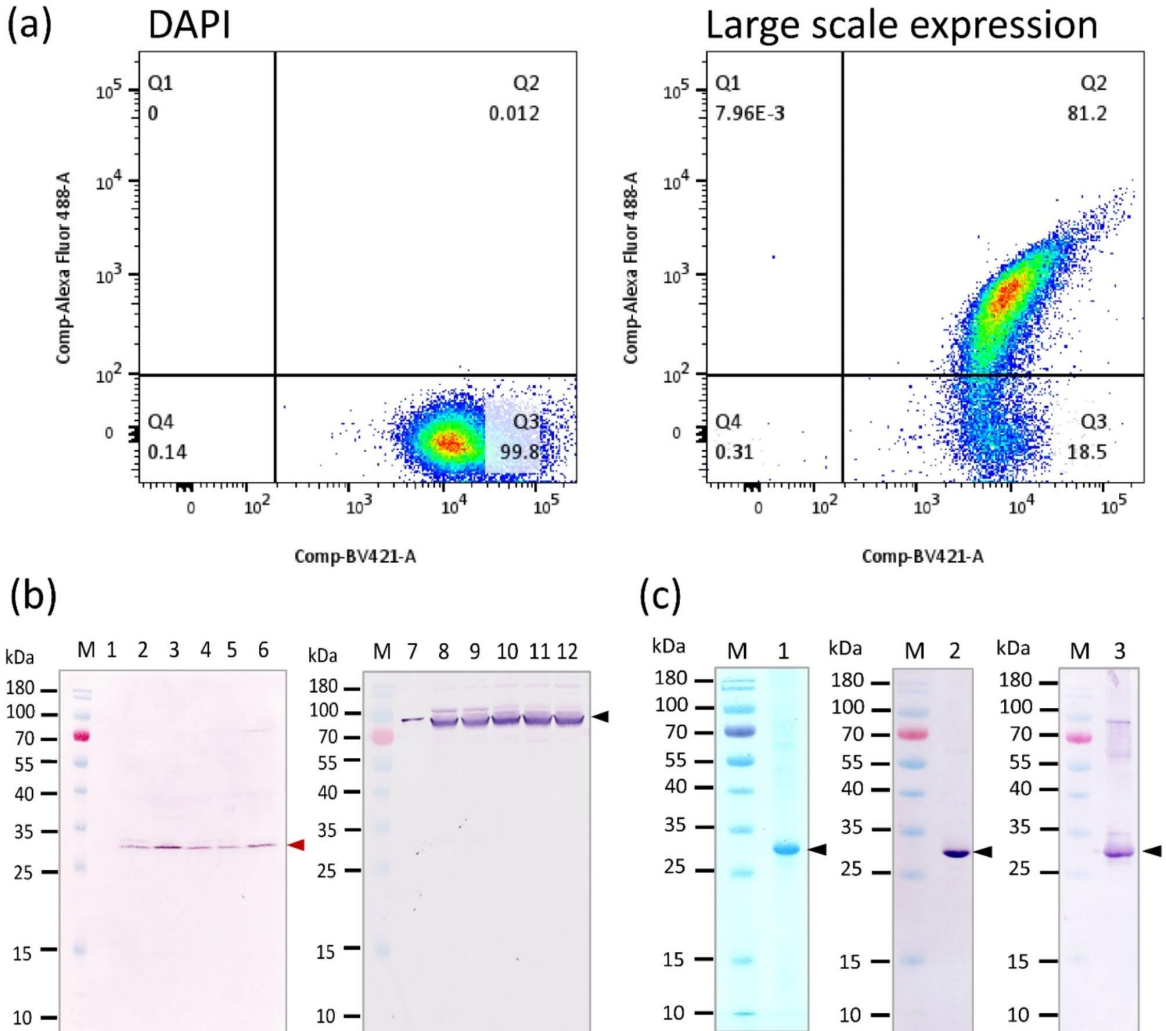
Large-scale culture of transformed BL 21 (DE3) ΔA *E. coli* for preparing G-PEN-HuscFv34-6× His. (**a**) The percentage of intact bacterial cells (grown in 1000 mL broth for 5 h) that displayed the fusion protein on their surface as determined by flow cytometry (Q2 of the right panel). (**b**) Left sheet: Western blot patterns and intensities of G-PEN-HuscFv34-6× His bands (between 25 and 35 kDa, arrowhead) in supernatants of the bacteria in (a) after incubating in Sortase cleavage buffer for 0, 1, 2, 3, 4 and 5 h (lanes 1–6, respectively). Right sheet, Western blot patterns of the proteins in the supernatants that were bound by anti-SUMO (arrowhead); this band should be SUMO-Δ59 Sortase-LPET- that still linked to LPP′-OmpA′ on the membrane of lysed bacterial cells. (**c**) Left sheet: Lane 1 is SDS-PAGE and CBB stained pattern of the purified G-PEN-HuscFv34-6× His (arrowhead) from the supernatant of the transformed bacteria suspended in Sortase cleavage buffer for 2 h; middle and right sheet show Western blot patterns of the purified G-PEN-HuscFV34-6× His probed with anti-His antibody (lane 2) and mouse monoclonal anti-HuscFv34 (lane 3). Lanes M of (b) and (c) are protein molecular masses in kDa

The transformed BL21 (DE3) ΔA competent *E. coli* was grown in large scale (400 mL LN-K broth) until OD 600 nm was 0.4–0.5. The bacteria were induced by 0.025 mM IPTG and incubated at 37 °C with shaking aeration (250 rpm) for 5 h. Figure 5a demonstrates the percentage of the bacterial cells that displayed the fusion protein on the surface (81.2%) after growing for 5 h. The bacterial cells were harvested from the culture, washed with 50 mM Tris (pH 7.5) containing 150 mM NaCl and resuspended with Sortase cleavage buffer (each 1 gram of bacterial wet weight was added with 10 mL of the buffer). The preparation was placed in 37 °C-water-bath for 5 h. The presence of G-PEN-HuscFv34-6× His in the supernatants at 0, 1, 2, 3, 4 and 5 h incubations were detected by Western blot analysis using mAb to HuscFv34 as the primary antibody. The G-PEN-HuscFv34-6× His could be released from the surface of the bacteria at every time point of incubation (except at 0 h) in the Sortase cleavage buffer; the highest yield was found at 2 h-incubation (Fig. 5b, bands located between 25 and 35 kDa markers, arrowhead). Therefore, the transformed BL21 (DE3) ΔA competent *E. coli* was grown in 1000 mL LN-K broth under 0.025 mM IPTG induction at 37 °C, 250 rpm; the harvested cells were washed with 50 mM Tris (pH 7.5) containing 150 mM NaCl, resuspended with Sortase cleavage buffer (1-gram bacterial wet weight per 10 mL buffer) and incubated in 37 °C-water-bath for 2 h. The G-PEN-HuscFv34-6× His purified from the supernatant was subjected to SDS-PAGE and CBB staining (Fig. 5c).

### Biocompatibility of the G-PEN-HuscFv34-6× His to mammalian cells

**Fig. 6:**
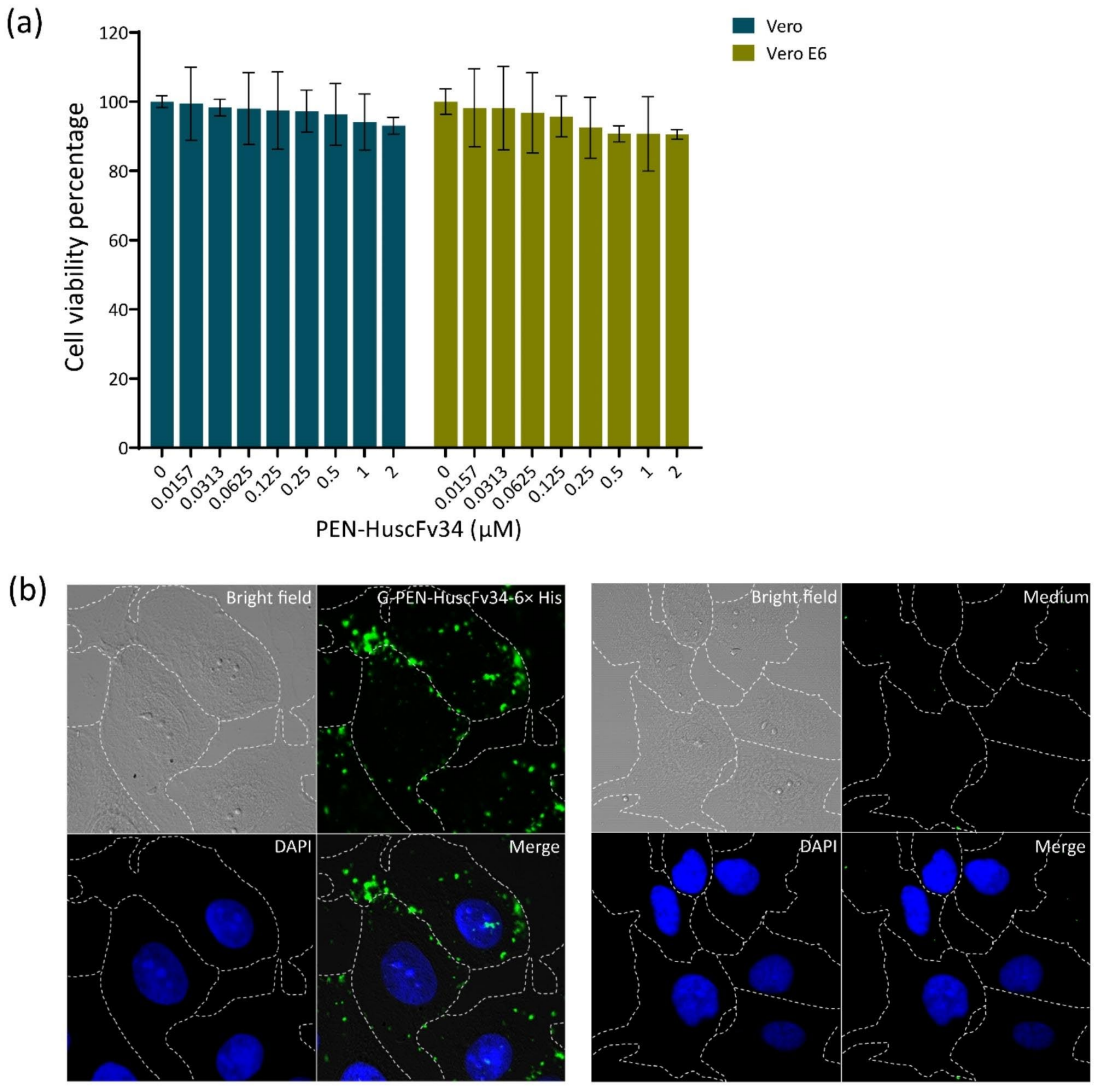
Biocompatibility and cell-penetrating ability of the G-PEN-HuscFv34-6× His. (**a**) Percent viability of cells: Vero (blue bars)/Vero E6 (olive bars) after treatment with different concentrations of G-PEN-HuscFv34-6× His at 37 °C in 5% CO_2_ incubator for 24 h. (**b**) Left block: Intracellular localization of the G-PEN-HuscFv34-6× His after adding 0.5 μM to the Vero E6 cells, incubated, fixed, permeated and probed with mAb to HuscFv34 followed by goat anti-mouse Ig-Alexa Fluor 488 and DAPI. The G-PEN-HuscFv34-6× His appears green; the nuclei stained blue; the cell contours are indicated by dotted lines. Right block, negative control (cells in medium instead of the G-PEN-HuscFv34-6x His and probed with mAb to HuscFv34)

Biocompatibility of the G-PEN-HuscFv34-6× His to Vero and Vero E6 cells (representative of mammalian cells) were determined by adding different concentrations of the G-PEN-HuscFv34-6× His (0–2.0 μM) to the cell monolayer and incubated at 37 °C in a 5% CO_2_ incubator overnight. Cytotoxicity of the G-PEN-HuscFv34-6× His was determined and compared to the cells in medium alone (no cytotoxicity). The calculated percentages of cell viability indicated that the G-PEN-HuscFv34-6× His was not toxic to the representative mammalian cells (Fig. 6a), i.e., Vero (blue bars) and Vero E6 (olive bars).

### Cell-penetrating ability of the G-PEN-HuscFv34-6× His

Vero E6 cells were incubated at 37 °C in 5% CO_2_ atmosphere with G-PEN-HuscFv34-6× His (0.5 μM) overnight. The cells were washed with PBS, fixed and permeated with ice-cooled methanol:acetone on ice, washed with PBS, blocked with 3% BSA in PBS, washed, and incubated with mAb to HuscFv34, washed, and added with goat anti-mouse Ig-Alexa Fluor 488 conjugate. DAPI was used to stain nuclei. The cells were mounted and observed under a confocal microscopy. The G-PEN-HuscFv34-6× His (green) was found in cytoplasm, indicating cell-penetrating ability (Fig. 6b) or being super antibody.

### Anti-RNA virus activity of the G-PEN-HuscFv34-6× His

**Fig. 7:**
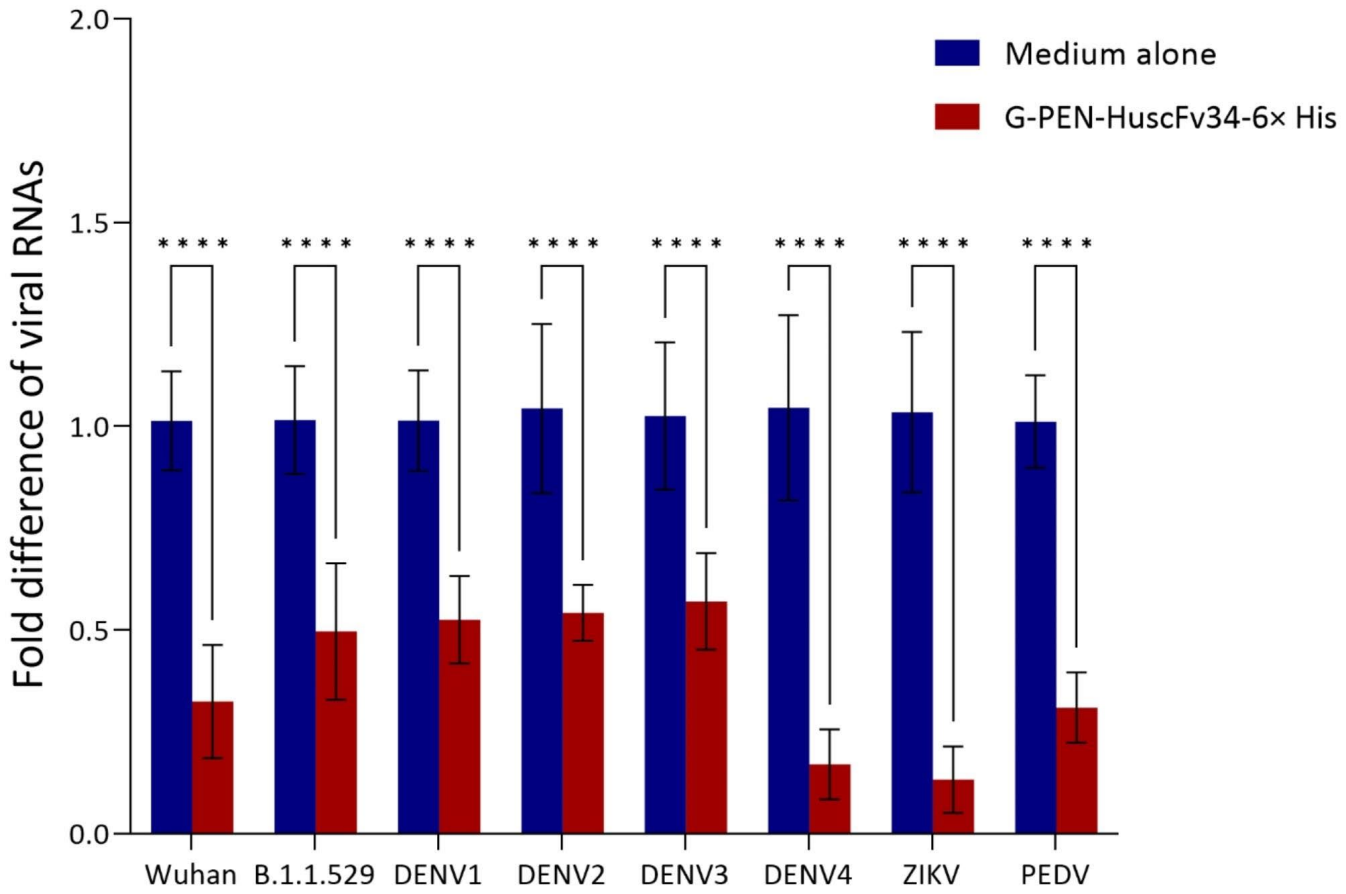
Fold-differences in viral RNAs recovered from G-PEN-HuscFv34-6× His treated-infected cells compared to medium treated-infected cells. Vero E6 cells were infected with *Betacoronavirus*, i.e., SARS-CoV-2 (Wuhan wildtype or B1.1.529 Omicron variant) while Vero cells were infected with flaviviruses (DENV serotypes 1–4, ZIKV) or *Alphacoronavirus* (PEDV) for 1 h. The fluids in wells were discarded and the infected cells were treated with G-PEN-HuscFv34-6× His (0.5 μM) for 18 h (SARS-CoV-2 and PEDV) or 48 h (DENV and ZIKV). Infected cells in medium alone served as controls. The cells were harvested for RNA extraction and the viral RNAs were quantified by qRT-PCR. ∗∗∗∗, *p* < 0.0001

Vero cells were added with Dengue virus (serotypes 1–4; DENV1, DENV2, DENV3 and DENV4), Zika virus (ZIKV) or PEDV; Vero E6 cells were added with SARS-CoV-2 (Wuhan wild type or BA 1. 1. 529 Omicron variant) and incubated for 1 h. After removing the supernatant from each well, medium containing 0.5 μM G-PEN-HuscFv34-6× His was added, and the treated cells were incubated for 18 h (SARS-CoV-2 and PEDV) or 48 h (DENV and ZIKV). The supernatants were discarded, and the cells were harvested for intracellular viral RNAs quantification by qRT-PCR. Data in Fig. 7 show the fold differences of the viral RNAs recovered from the infected cells treated with G-PEN-HuscFv34-6× His (super antibody) compared to the viral RNAs of the respective virus-infected cells in culture medium alone (negative inhibition control). The G-PEN-HuscFv34-6× His could significantly reduce the intracellular RNAs of the viruses that were tested compared to the infected cells in medium alone (*p* < 0.0001).

## Discussion

During the past few decades, several RNA viruses have emerged or re-emerged to cause diseases that threaten global human/animal health and socioeconomics, such as H5N1 and H1N12009pdm influenza viruses [21, 22] Dengue viruses [23], Zika virus [24], Ebola virus [25], Marburg virus [26], Enterovirus 71 [27], human and animal coronaviruses including SARS-CoV, MERS-CoV, Porcine Epidemic Diarrhea virus (PEDV) [28, 29], SARS-CoV-2 [30] and others. Several virus infections have no preventable vaccines, or the available vaccines are unsatisfactorily effective in preventing re-infections and may cause undesirable side effects/sequels. Effective and safe vaccines as well as drugs/therapeutics are needed for prevention and treatment of the deadly virus-causing diseases.

RNA-dependent RNA polymerase (RdRp) is a versatile enzyme that is pivotal for genome replication, mRNA synthesis and virus protein production for virus fitness, host immunity evasion/suppression, drug resistance and pathogenesis. The molecular architecture of the virus RdRp proteins is common; they all acquire a unique encircled right-hand architecture with the catalytic site surrounded by palm, fingers, and thumb domains [31, 32]. The RdRp proteins of certain RNA viruses may have additional domains, like methyltransferase, helicase, NTPase or endonuclease, and associated host proteins, e.g., translation factors, protein chaperones, RNA-modifying enzymes, which the viruses exploit for attaining enhanced RdRp activity in the virus RNA synthesis [33–36]. RNA dependent RNA polymerase of several RNA viruses including those of Family Flaviviridae (Hepatitis C virus, Dengue, Zika, Japanese encephalitis and West Nile viruses); Family Picornaviridae (e.g., enteroviruses, polioviruses); Family Coronaviridae (e.g., SARS-CoV, MERS-CoV, SARS-CoV-2 and PEDV) and others, are highly conserved at the amino acid level in the catalytic site and lack human homolog. Thus, drugs/small chemicals [37, 38] or antibodies [5, 6] that inhibit RdRp activity should be broadly effective against most, if not all, RNA viruses and should be relatively safe for human treatment.

Antibody-based treatments have been used to treat diseases for several decades [39]. For virus infections, most therapeutic antibodies work extracellularly and direct against virus infectivity, i.e., neutralizing antibodies prevent cellular entry by block receptor binding, or other effector functions, e.g., antibodies mark the virus-infected cells for destruction by immune cells and/or complement products [40]. Contemporary biotechnologies including genetic engineering, recombinant protein production, imaging technologies and many others have revolutionized production methods and formats as well as anti-viral mechanisms of engineered antibodies that are used in immunotherapies. Recently, we generated an engineered super antibody against RdRp (NS5B) of hepatitis C virus in the form of cell-penetrating human single-chain antibody (HuscFv) by phage display technology [5]; HuscFv of one phage infected *E. coli* clone (clone 34) was linked to a cell-penetrating peptide, penetratin (PEN) to form (PEN-HuscFv34) or transbody/super antibody [5]. The term “Super antibody” was coined by Charles Morgan, president of InNexus Biotechnology Inc, Vancouver, Canada, which has developed the super-antibody technology, for the antibodies that can bind to target inside the cells [41]. Super antibody is a new generation of broadly effective and/or highly potent monoclonal antibodies [42]. Super antibodies enter cells; if no target inside the cells, they come out and enter other cells until they find the intracellular target which then they bind, and subsequently the immune complexes are eliminated physiologically by cellular machineries, e.g., ubiquitin proteasome [43], autophagy [44] and/or TRIM21 system [45]. The super antibody to virus RdRp (PEN-HuscFv34) that was generated not only inhibited replication of the homologous hepatitis C virus (HCV) and restored the innate anti-virus activity that had been suppressed by the HCV [5], but also could inhibit replication of other RNA viruses of the same Flaviviridae family (Dengue and Zika viruses) and viruses of other families including *Alphacoronavirus* (PEDV), *Betacoronavirus* (SARS-CoV-2 wild type and variants of concern) and Picornaviridae (EV71) that were tested [6]. Thus, the broadly effective super antibody has high potential for developing further towards a pan-anti-RNA virus agent.

*Escherichia coli* has been used extensively as a microbial cell factory for synthesis of heterologous proteins for research or therapeutic applications by recombinant DNA technology. Currently, there is a wealth of *E. coli* mutants that are well suited for fast growth, simple manipulation, cost-effectiveness, and readiness for scaling-up the recombinant proteins [46]. Also, there are associated tools, such as plasmids and cultivation conditions to optimize the product yield [46]. High-level of the mature heterologous protein expression up to 35% of total cellular proteins could be obtained from transformed *E. coli* [47]. Nevertheless, there are still bottlenecks of foreign protein production in the *E. coli* system. Besides lack of glycosylation which may be required for certain recombinant proteins, the overexpressed proteins tend to be in the *E. coli* insoluble aggregates, called bacterial inclusion bodies (IB) [48]. Thus, to obtain soluble and functionalized proteins, usually the IBs must be purified and solubilized in chaotropic co-solutes that can disrupt the hydrogen bonding network for weakening the hydrophobic effect between molecules which, in the effect, could reduce stability of the native/near native state of the recombinant proteins. Also, this process may result in the protein misfolding, hence the loss of their functional activities, on top of the low product yield [49]. Complicate and time-consuming downstream process is required to produce soluble proteins from the *E. coli* aggregates including bacterial cell breaking, isolation and purification of the IBs, and protein refolding which are hurdles for large scale production of the proteins especially the industrialized commercial goods, such as therapeutic proteins and antibodies. Therefore, in this study, recently developed bacteria surface display and Sortase self-cleave systems [7, 8] were adopted with additional modification to suit our super antibody production in soluble, naturally folding form for future up-scaling production.

The BL21 (DE3) **Δ**A competent *E. coli* strain was used as a plasmid-transformed competent host for displaying our designed fusion protein: Lpp′-OmpA′-SUMO-Δ59 Sortase A-Sortase cleavage motif (LPETG)-PEN-HuscFv34-6× His. This bacterial strain is a derivative of B line BL21 (DE3) *E. coli* (Thermo Scientific) that lacks gene coding for predominant outer membrane protein, OmpA. The bacteria are deficient in two proteases, i.e., ion protease and outer membrane protease, OmpT. The lack of these proteases reduces degradation of heterologous proteins expressed in the *E. coli* cells (Thermo Scientific). Besides, this *E. coli* strain contains λDE3 prophage that carries the gene for T7 RNA polymerase under control of a *lac*UV5 promoter, allowing expression of the T7 RNA polymerase that can be induced with isopropyl β-D-1-thiogalactopyranoside (IPTG), a highly stable compound that molecularly mimic allolactose (a lactose metabolite) that triggers transcription of the *lac* operon (Thermo Scientific). The modified OmpA protein called chimeric LPP′-OmpA′ developed by Georgiou et al. [50] was used as a trans peptidoglycan-outer membrane linker for serving as a vehicle to surface-display our passenger fusion domains (SUMO-Δ59 Sortase A-LPETG motif-PEN-HuscFv34-6× His) [51]. The chimeric LPP′-OmpA′ protein consists of a signal peptide and the first nine residues of an abundant lipoprotein of *E. coli* called Braun’s lipoprotein (BLP) (murein lipoprotein or Lpp that is responsible for tethering the inner membrane peptidoglycan to the outer membrane of *E. coli*), linked to five of the eight membrane-spanning segments of the OmpA porin (residues 46–159). The Lpp amino terminus (Lpp′) in the Lpp′-OmpA′ construct targets passenger proteins to the outer membrane for displaying on the *E. coli* surface [50, 51].

In 2004, Mao developed a new self-cleavable Sortase fusion system to generate free recombinant protein that could be purified by a single-step affinity column chromatography [8]. A recombinant fusion protein consisted of an *N*-terminal 6× His tag fused with catalytic core of Sortase A from *Staphylococcus aureus* (SrtAc), followed by LPETG (Sortase cleavage motif) and protein of interest at the C-terminus, was expressed in a transformed *E. coli* [8]. The *E. coli*-derived fusion protein was then immobilized on the ion metal- affinity chromatography (IMAC) resin. In the presence of Ca^2+^ and/or triglycine, the Sortase cleaved the immobilized fusion protein at the LPET↓G site from where the target protein with an extra *N*-terminal glycine (G) is released, leaving behind the *N*-terminal portion that remained bound to the column resin. This approach is simple, robust, inexpensive, time saving, and allows purification of free recombinant protein via one-step chromatography [8]. In this study, we have combined the activity of the chimeric Lpp′-OmpA′ and the Sortase self-cleave fusion system to display our fusion protein on the *E. coli* surface, such that the recombinant protein isolation from the bacteria and the IMAC step [8] could be omitted and the protein of our interest (PEN-HuscFv34 to RNA virus RdRp) acquired a free-folding structure. Besides the Sortase catalytic module and the Sortase cleavage motif on the fusion protein, a small (100 amino acids) protein named small-ubiquitin-like modifier (SUMO) was linked at its *N*-terminus to the Lpp′-OmpA′ followed by the Sortase-Sotrase cleavage site-PEN-HuscFv34-6× His. SUMO has been known as the solubility and expression-enhancing tag used for recombinant protein expression; the SUMO fusion tag has been used frequently as an *N*-fusion sequence in recombinant protein production in both prokaryotic and eukaryotic system [52]. In this study, optimal condition in growing the transformed *E. coli* was investigated including IPTG concentration and temperature, as well as the bacterial wet weight to the Sortase cleavage buffer ratio and time of incubation to release the target protein from the bacterial surface, which was then purified by one-step affinity HisTrap HP column chromatography. The purified G-PEN-HuscFv34-6× His was not toxic to mammalian cells, traverse across the cell membrane and inhibited replication of RNA viruses that were tested.

## Conclusion

The G-PEN-HuscFv34-6× His produced by the combined bacterial surface display and the Sortase self-cleave systems with modifications in this study retained the cell-penetrating ability (being super antibody) and the pan anti-RNA virus activity of the PEN-HuscFv34 produced previously from the *E. coli* inclusion body [5, 6]. The display system should be suitable for downstream process in a large-scale production of the super antibody. It is applicable also to produce other recombinant proteins in soluble, free-folding form.

### Electronic supplementary material

Below is the link to the electronic supplementary material.

**Additional file 1: Table S1**. Results of qRT-PCR for determination of virus RNAs recovered from virus infected cells treated with G-PEN-HuscFv34 and infected cells in medium alone

## Data Availability

All data generated or analyzed in this study are included in this article and its additional file.

## Abbreviations

AP: Alkaline phosphatase
BCIP/NBT: 5-bromo-4-chloro-3-indolyl phosphate/nitro blue tetrazolium
CBB: Coomassie Brilliant Blue G 250
CPP: Cell-penetrating peptide
DAPI: 4′,6-diamidino-2-phenylindole
DENV: Dengue virus
DMEM: Dulbecco’s modified Eagle’s medium
DNA: Deoxyribonucleic acid
DPBS: Dulbecco’s phosphate-buffered saline
DTT: Dithiothreitol
*E. coli*: *Escherichia coli*
FACS: Fluorescence-activated cell sorting
FBS: Fetal bovine serum
HCV: Hepatitis C virus
HuscFv: Human single-chain antibody variable fragment
IB: Inclusion body
IMAC: Ion metal- affinity chromatography
IPTG: Isopropyl-β-D-thiogalactoside
LB: Luria-Bertani
LN: Lennox
Lpp′: A portion (a signal peptide and the first nine residues) of Braun’s lipoprotein (BLP)
mAbs: Monoclonal antibodies
MERS-CoV: Middle East Respiratory Syndrome coronavirus
NC: Nitrocellulose membrane
NTPase: Nucleoside-triphosphatase
OmpA: Membrane-spanning outer membrane protein A
OmpA′: Residues 46–159 of OmpA
PBS: Phosphate-buffered saline, pH 7.4
PEDV: Porcine Epidemic Diarrhea virus
PEN: Penetratin
qRT-PCR: Real-time (quantitative) reverse transcription-polymerase chain reaction
RdRp: RNA-dependent RNA polymerase
RNA: Ribonucleic acid
RT: Room temperature (25 ± 2 °C)
SARS-CoV: Severe Acute Respiratory Syndrome coronavirus
SDS-PAGE: Sodium dodecyl sulfate-polyacrylamide gel electrophoresis
SUMO: Small-ubiquitin-like modifier protein
TBS-T: Tris-buffered saline containing 0.01% polysorbate (Tween)-20 nonionic detergent
TPCK: Tosyl phenylalanyl chloromethyl ketone
Tris: Tris (hydroxymethyl) aminomethane (C_4_H_11_NO_3_)
ZIKV: Zika virus
